# CD9 and folate receptor overexpression are not sufficient for VSV-G-independent lentiviral transduction

**DOI:** 10.1371/journal.pone.0264642

**Published:** 2022-03-10

**Authors:** Cristina Bellotti, Andreas Stäuble, Robert Steinfeld

**Affiliations:** Department of Paediatric Neurology, University Children’s Hospital Zurich, University of Zurich, Zurich, Switzerland; Arizona State University, UNITED STATES

## Abstract

Extracellular vesicles have become a research focus for their potential as therapeutic vehicles that carry cargo substances. Extracellular vesicles may origin from the endosomal compartment and share several characteristics with the envelope of lentiviruses. A previous study reported that constitutive expression of the tetraspanin CD9, an extracellular vesicle marker, not only increases vesicle secretion from cells, but has also a positive effect on lentiviral transduction efficiency. Moreover, it was shown that expression of CD9 on the viral envelope in absence of viral glycoproteins was sufficient for the transduction of mammalian cells. In this study, we investigate the effect of CD9 and folate receptor alpha, a GPI-anchored protein, on biosynthesis and transduction efficiency of vesicles carrying lentiviral vectors. We demonstrate that neither CD9 nor FRα nor the combination of both were able to mediate a significant transduction of therapeutic vesicles carrying lentiviral RNA. Further studies are required to identify endogenous mammalian proteins that can be used for pseudotyping of viral envelopes to improve viral targeting without inducing immune responses.

## Introduction

Extracellular vesicles (EVs) are defined as “particles naturally released from the cell that are delimited by a lipid bilayer and cannot replicate” [1]. They can be of endosomal origin (traditionally called “exosomes” [2]) or plasma-membrane derived (“ectosomes”) [3]. Selectively isolating one of these subpopulations is particularly challenging, as they share similar physical characteristics and protein markers [4] but may be achieved by the combination of advanced purification procedures. Exosomes share several characteristics with lentiviral particles: they both have a lipid bilayer membrane, they are similar in size and density [5, 6] and they carry nucleic acids such as RNA [7]. In addition, one of the main pathways of exosome biogenesis is driven by the endosomal sorting complex required for transport (ESCRT-dependent pathway) [8, 9] and some ESCRT-associated proteins, like Alix and tumor susceptibility gene 101 protein (TSG101), are considered exosomal markers [10–12]. ESCRT, Alix and TSG101 are all involved in virus budding [13–15]. Such similarity in the formation process of exosomes and lentivirus (LV) prompted some scientists to propose the Trojan exosome hypothesis, which states that LVs hijacked the pre-existing cellular pathway of exosome biogenesis for infectious particles production [16]. In general, EVs produced by virus-infected cells can contain viral proteins [17, 18] and RNA [19, 20] and play a role in infection [21].

HIV-based LVs are one of the most used vectors for gene-therapy studies, and one of their advantages is the possibility to manipulate their tropism by changing the glycoproteins expressed on their envelope (a process called pseudotyping) [22]. Vesicular stomatitis virus GP (VSV-G) has become the standard for pseudotyping because it allows concentration of vectors to a high titre and it enables infection of a vast range of cell types [23]. However, using VSV-G has its disadvantages: if used in human subjects, it can elicit adaptive immune responses that cause vector inactivation [24]. This could result in major immune reactions if the therapy requires multiple application of the vector. Solutions to this problem are a focus of research: for example, it was proposed that the use of heterologous G-proteins from different viruses of the vesiculovirus genus in subsequent vector injections might circumvent the adaptive immune response [25].

Recently, EVs and in particular exosomes have become subjects of research for their potential clinical applications as diagnostic markers and as vectors in targeted drug delivery [26, 27]. The advantages of using EVs as drug carriers are their broad immune tolerance, low toxicity, good tissue penetrance and easy engineerability. In particular, since EVs are purified from mammalian cells they express only endogenous proteins that may not induce any immune response.

Addressing the relationship between exosomal and lentiviral biogenesis, a study in 2018 investigated the effect of constitutive expression of exosomal markers on EVs secretion and LV production and infectivity [28]. One of the tested proteins was CD9, belonging to the tetraspanins, which are proteins commonly expressed on EVs [29]. This study demonstrated that overexpression of CD9 caused an increase in EVs production and an improvement of LV efficiency in gene delivery. The most notable result, though, was that CD9 expression allowed to achieve LV transduction in the absence of pseudotyping with the viral glycoprotein VSV-G.

The possibility to engineer transduction-efficient LV vectors without viral envelope proteins would represent a major advantage, even if the infection rate was lower than in the presence of VSV-G. The fact that CD9 is an endogenous human protein means that it would completely avoid the development of adaptive immunity.

Based on these premises, we asked ourselves whether it was possible to use CD9 in conjugation with other EVs markers to further functionalize the LV vectors and increase specific targeting to certain tissues and cells. Folate receptor alpha (FRα) is a protein expressed by epithelial cells derived from kidney, lung and breast [30]. Most importantly, FRα is expressed in choroid plexus cells and was identified as the main folate transporter to the central nervous system (CNS) [31, 32]. It is anchored to the membrane by a glycosylphosphatidylinositol (GPI) anchor [33] and it is transported to GPI-enriched early endosomal compartments [34]. Although FRα is not classically considered an EVs marker, it was found to be expressed on the surface of exosomes secreted by the choroid plexus cells into the cerebrospinal fluid (CSF) [32]. In addition, we demonstrated that histidine-tagged FRα can be used as a marker to selectively purify EVs of endosomal origin with a chromatographic approach [35]. Since FRα-positive vesicles in the cerebrospinal fluid are able to cross the ependymal cell layer and deliver folate to the brain parenchyma, it was hypothesized that FRα could be used to increase delivery of therapeutics to the brain [32]. In this study, we examined the influence of constitutive expression of FRα in conjugation with CD9 on EVs production and LVs infectivity. Our goal was to obtain CNS-targeted transduction-efficient vectors for clinical application in cases of neurodegenerative diseases.

## Material and methods

### Cell culture

All cells were grown in a humidified incubator at 37°C and 5% CO_2_. All cell lines were cultured in Dulbecco’s Modified Eagle’s Medium (DMEM) + GlutaMAX (Gibco) with 10% Fetal Bovine Serum (FBS) and appropriate antibiotics. For experiments involving EVs isolation, cell lines were adapted to Serum Free Medium 293 (SFM II) (Gibco). First, a ratio of 1:1 DMEM to SFM supplemented with GlutaMAX (Gibco) was used. Then SFM percentage was gradually increased until cells could survive in 100% SFM.

### Plasmids

pCMV-VSV-G and pLenti6.3-CD9_GFP_ were a gift from J. Gruber, German Primate Center Göttingen, Germany. pCMV-dR8.91 and SEW_SFFVU3_GFP were a gift from J. Reichenbach, University of Zurich, Switzerland.

The CD9 sequence was amplified from cDNA of HEK 293 cells (primer sequences are listed in S1 Table). NheI and XhoI restriction sites were attached at the N- and C-terminus in a second PCR step so that the sequence could be inserted in a modified version of the pBlueScript II plasmid. The CD9 sequence was cut out and inserted into pCDNA-IRES-T3, while a copy of N-terminally polyhistidine-tagged FRα was inserted into pEF-T1. Both plasmids were then cut with NotI and PvuI and ligated together to obtain a single plasmid carrying the sequence of both target genes (pEFTT-HisFRα-CD9).

To produce pLenti6.3-CD9 the GFP-CD9 insert was cut out from pLenti6.3-CD9_GFP_ and replaced with the CD9 sequence. For pLenti6.3-EGFP, the EGFP sequence was cut out from SEW_SFFVU3_GFP and inserted into the pLenti backbone.

All PCR steps were performed using the Phusion High-Fidelity PCR Kit (New England BioLabs) and were carried out in a TProfessional Thermocycler (Biometra Ltd). Oligonucleotide production and sequencing were done by Microsynth AG.

### Generation of stable cell lines

Human embryonic kidney HEK 293 cells were purchased from ATCC. HEK 293T were a gift from J. Reichenbach, University of Zurich, Switzerland.

The FRα overexpressing cell line was generated as previously described [35]. To generate the FRα/CD9 overexpressing line 300,000 HEK 293 cells per well were plated in a 6-well plate and cultured for about 7 hours. Calcium-phosphate precipitation was then used to transfect the cells with 1 μg pEFTT-HisFRα-CD9. Selection was performed adding 1 μg/ml Puromycin (Gibco) and 50 μg/ml Geneticin (Gibco) to the media.

To generate the CD9 and GFP-CD9 cell lines, LVs carrying pLenti6.3-CD9 or pLenti6.3-CD9_GFP_ were produced in HEK 293T. 50,000 HEK 293T were then transduced with a 1:10 dilution of one of these LVs. After 70 h incubation, the virus was removed and cells were selected with 10 μg/ml Blasticidin (Gibco).

### Protein extraction and quantification

Cell pellets were collected, washed in PBS, resuspended in lysis buffer (50 mM Tris-HCl pH 7.4, 150 mM NaCl, 1 mM EDTA, 0.5% Sodium deoxycholate, 0.1% SDS) containing proteinase inhibitors, incubated on ice for 15 minutes and centrifuged for 15 min, 14500 rpm at 4°C. The resulting supernatant was collected and protein concentration was estimated with a Pierce BCA assay using the Pierce BCA Protein Assay Kit (Thermo Fisher Scientific).

### Western Blot (WB)

All antibodies were diluted in blocking solution: Tris Buffered Saline + 1% Tween (TBST) containing 3% Bovine Serum Albumin (BSA, Sigma-Aldrich). Working dilutions: anti-FRα 1:20,000 (NCL-L-FRalpha, Leica Biosystems), anti-CD9 1:1,000 (ab92726, Abcam), anti-AIP1/Alix 1:1,000 (ABC40, Merck Millipore), anti-HSP 90α/β 1:75,000 (sc-13119, Santa Cruz), Goat Anti-Rabbit IgG H&L (HRP-conjugated) 1:5,000 (ab6721, Abcam), Goat Anti-Mouse IgG H&L (HRP-conjugated) 1:5,000 (ab97023, Abcam). 8 to 15 μg protein per sample were diluted in 4x Laemmli Sample Buffer (Bio-Rad Laboratories), incubated for 5 min at 95°C and loaded on an acrylamide gel. Transfer to a 0.2 μm PVDF membrane was performed in a Tran-Blot Turbo Transfer System (Bio-Rad Laboratories) using a Trans-Blot Turbo RTA Mini Transfer Kit (Bio-Rad Laboratories). Membranes were incubated in blocking solution for at least 30 min room temperature (RT) and then in the primary antibody solution overnight at 4°C. The following day the membranes were washed 4 times in TBST and incubated in the secondary antibody solution for 2 hours at RT. Afterwards, washings in TBST were repeated. A 1:1 mix of the Clarity ECL Western Blotting Substrates (Bio-Rad Laboratories) was applied to the membranes and signal was registered using a ChemiDoc XRS+ (Bio-Rad Laboratories). Western Blot images were analysed using the Image Lab software (Bio-Rad Laboratories).

### RNA extraction and Reverse-Transcription-qPCR

RNA from 3x10^6^ cells was extracted with the RNeasy Mini Kit (Qiagen) and eluted in 50 μl H_2_O. RNA concentration was measured using a NanoDrop Lite Spectrophotometer (Thermo Fisher Scientific). To eliminate potential contaminations from genomic DNA, 10 μg RNA were incubated with 2 U DNase I (New England BioLabs) at 37°C for 30 min. The RNA was then cleaned again using the RNeasy Mini Kit and finally eluted in 30 μl H_2_O. RNA quality was checked by running 500 ng per sample on a 1% agarose gel. 1 μg of the resulting RNA was reverse transcribed using the ReadyScript cDNA Synthesis Mix (Sigma-Aldrich) per manufacturer instructions. 1 μl of the reaction was used as template in the qPCR with the Luna Universal qPCR Master Mix (New England BioLabs) and a CFX96 Real-Time PCR Detection System (Bio-Rad Laboratories). Three PCR replicates were made per sample. Relative expression of CD9 was calculated via the ΔΔCT-method as the housekeeping gene. Primers sequences were taken from Boker and colleagues (2018) [28].

### Viral production

6-well plates were incubated with Poly-L-Ornithine Solution (0.01%) (Merck Millipore) for at least 1 hour at 37°C before cell seeding. 600,000 cells per well from the appropriate cell lines were seeded 24 hours before transfection. Transfection was achieved by applying a DNA: polyethylenimine (PEI) 1:3 (w/w ratio) mixture containing 0.21 pmol transfer plasmid carrying the gene of interest, 0.17 pmol packaging plasmid (pCMV-dR8.91) and (if needed) 0.09 pmol envelope plasmid (pCMV-VSV-G) to the cells. Media was changed after overnight incubation. After approximately 55 hours incubation, virus-containing supernatant was harvested, centrifuged at 1,000 rpm for 5 min and filtered through a 0.4 μm filter to eliminate cells and debris, snap-frozen and stored at -80°C. LVs titration was performed using the Lenti-X qRT-PCR Titration Kit (Takara Bio Inc.) following manufacturer’s instructions. The qRT-PCR was done in a CFX96 Real-Time PCR Detection System (Bio-Rad Laboratories) and data was analysed using the CFX Maestro software (Bio-Rad Laboratories).

### Lentiviral transduction

For analysis with flow cytometry, 50,000 HEK 293T cell (P<25) were transduced with a viral titre of 10^6^/10^7^ in a 24-well plate. 48 hours after transduction, cells were harvested, fixed with 2% formaldehyde for 10 minutes, washed with Phosphate-buffered saline (PBS) and resuspended in PBS with 2% FBS and 0.1% Sodium azide. A BD LSRFortessa Cell Analyzer (BD Biosciences) was used to record at least 5,000 events per sample. The FITC LP505-BP530/30 filter set was used for GFP detection. Data were recorded using the FACSDiva Software (BD Biosciences) and further analysed with FlowJo (BD Biosciences).

For microscopy analysis, 25,000 HEK 293T cell (P<25) per well were transduced with a viral titre of 10^6^/10^7^ in a 24-well plate. 72 hours after transduction cells were stained with Hoechst 33342 1:2,000 (Thermo Fisher Scientific) and imaged with an Axio Observer Z1 (Zeiss). 3 images per well were acquired using the 20X objective and a Hamamatsu Orca Flash 4.0 camera. Laser intensity and exposure time were kept constant for the whole experiment.

All transductions were done in duplicates.

### EVs markers analysis

Cells adapted to SFM were centrifuged at 1,000 rpm for 5 min. 5–7 ml of supernatant were harvested and further centrifuged at 2,000 g for 30 min to remove debris, mixed with 0.5 volumes of Total Exosome Isolation (from cell culture media) reagent (Invitrogen) and processed according to the manufacturer’s instructions. EVs were finally resuspended in 100 μl PBS and protein concentration was estimated with a BCA assay.

For subsequent WB analysis, a sample volume equivalent to 12.5 μg protein content was diluted in Isolation Buffer (PBS 0.1% BSA, 0.2 μm filtered), mixed with Exosome-Human CD9 Isolation Reagent (from cell culture) (Thermo Fisher Scientific) or Exosome-Human CD63 Isolation/Detection Reagent (from cell culture media) (Thermo Fisher Scientific) and processed as per manufacturer’s instructions. At the end of the protocol, samples were resuspended in lysis buffer and further prepared for loading (see above).

For flow cytometry, a sample volume equivalent to 25 μg protein content was diluted in Isolation Buffer (PBS 0.1% BSA, 0.2 μm filtered), mixed with Exosome-Human CD9 Isolation Reagent (from cell culture) (Thermo Fisher Scientific, diluted 5 times) or Exosome-Human CD63 Isolation/Detection Reagent (from cell culture media) (Thermo Fisher Scientific) and processed as per manufacturer’s instructions. Beads-bound exosomes were resuspended in 300 μl Isolation Buffer. 100 μl of each sample were then incubated with APC/Fire 750 anti-human CD9 Antibody 1:100 (312113, BioLegend) and PE anti-FOLR1 (Folate Binding Protein) Antibody 1:50 (908303, BioLegend) for 1h on a shaker (1,000 rpm) at RT. Samples were washed twice, resuspended in 300 μl Isolation Buffer and analysed with a BD LSRFortessa Cell Analyzer (BD Biosciences). The PE LP555-BP582/15, APC-Cy7 LP735-BP780/60 and FITC LP505-BP530/30 filter sets were used. At least 5,000 events per sample were recorded using the FACSDiva Software (BD Biosciences) and further analysed with FlowJo (BD Biosciences). Beads-only samples were used as negative controls.

### EVs production test

200,000 cells from the WT/FRα/FRα+CD9 lines adapted to SFM were seeded in 6-well plates (3 technical replicates per line). Cells were pelleted by centrifugation (1,000 rpm for 5 min) and the supernatant was further centrifuged at 2,000 g for 30 min to remove debris. Supernatant was then mixed with 0.5 volumes of Total Exosome Isolation reagent and samples were further processed as per manufacturer’s instructions. Final resuspension of EVs pellets was in 200 μl PBS. EVs concentration was measured with the ZetaView TWIN (Particle Metrix GmbH) using the 488 nm laser in scatter modality. Sensitivity was set to 80%, shutter to 100 units and temperature to 24°C. Samples were diluted 1:50 to 1:200 in PBS and recorded in triplicates (Measurement Mode: Size Distribution, 3 Cycles, 11 Positions). EVs parameters were calculated by the ZetaView software (Particle Metrix GmbH).

### Statistical analysis

Statistical analysis was performed using GraphPad Prism 8 (GraphPad Software). Significance of difference between experimental groups was estimated using post-hoc tests of ANOVA (details about the analysis are specified in the result section for each experiment). The threshold to accept statistical significance was set at alpha level 0.05 for all p-values.

## Results

### Establishment of CD9-overexpressing lines

**Fig 1:**
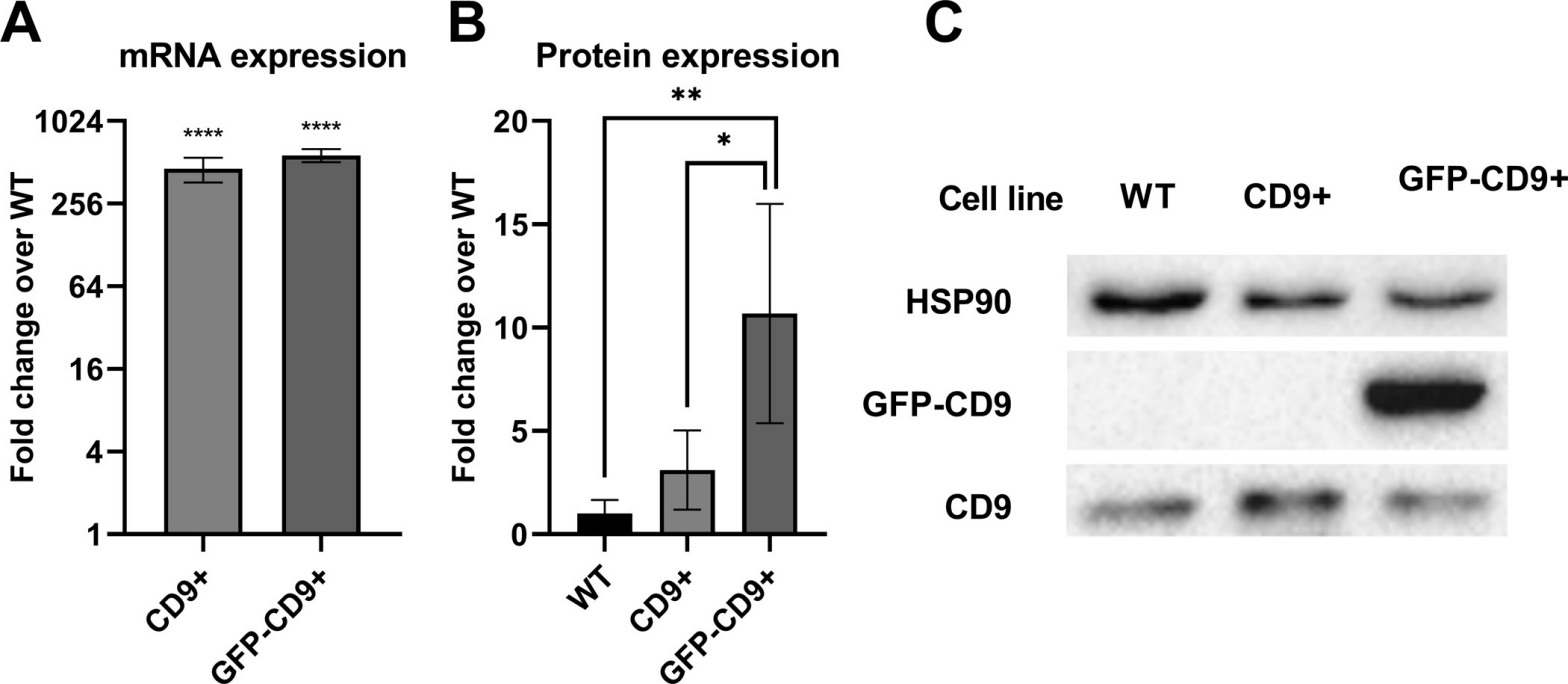
Change of CD9 expression in CD9+ and GFP-CD9+ lines. (A) Expression of CD9 mRNA was estimated by qPCR. Fold change data are represented as mean ± s.d. in log_2_ scale (n = 3 for each line). (B) CD9 protein expression was estimated by WB. Band intensity was normalized over the loading control HSP90. All data were normalized over the average of the WT group and are shown as mean ± s.d. (n = 4 for each cell line). (C) Example of WB membrane stained for CD9 and HSP90 as loading control. Note that samples from the GFP-CD9+ cell line present 2 bands when anti-CD9 antibody is used: one band correspond to the endogenous protein, the other to the fusion GFP-CD9 protein. * = p≤0.05; ** = p≤0.01; **** = p≤0.0001.

To replicate experiments about CD9 effect on transduction efficiency, we established two cell lines that overexpressed CD9. Using the same transfer plasmid (pLenti6.3-CD9_GFP_) to generate one of the two cell lines, we provided very similar conditions as previously reported [28]. The other line was created by transduction with pLenti6.3-CD9 to exclude any possible GFP transfer from the fusion protein. CD9 expression was tested with qPCR and WB. qPCR results indicate a ΔΔC_q_ of -8.82±0.32 s.d. for the CD9+ line and -9.17±0.15 s.d. for the GFP-CD9+ line compared to the average of the WT samples (0± 0.02 s.d.), equivalent to a change in expression of 459.33 and 576.44 times respectively (Fig 1A). p was <0.0001 in both cases when comparing the ΔΔC_q_ of the experimental lines to WT cells (Tukey’s multiple comparisons test post ordinary one-way ANOVA). There was no significant difference between the CD9+ and GFP-CD9+ lines. However, this huge increase in mRNA synthesis was not translated in a corresponding protein overexpression. When expression levels were estimated via WB, in fact, the overexpression was only of 3.11± 1.91 s.d. times for the CD9+ line and of 10.68± 5.31 s.d. times for the GFP-CD9+ line (Fig 1B and 1C). Moreover, while there was a significant difference between WT (1.00± 0.67 s.d.) and GFP-CD9+ line (p = 0.0061, Tukey’s multiple comparisons test post ordinary one-way ANOVA), the difference between WT and CD9+ cells was not significant. There was instead a significant (p = 0.0239) difference between protein expression in the CD9+ and the GFP-CD9+ line.

As previously reported [28], expression of the fusion protein GFP-CD9 was localized to the cell membrane (S1 Fig).

### Characterization of EVs

**Fig 2:**
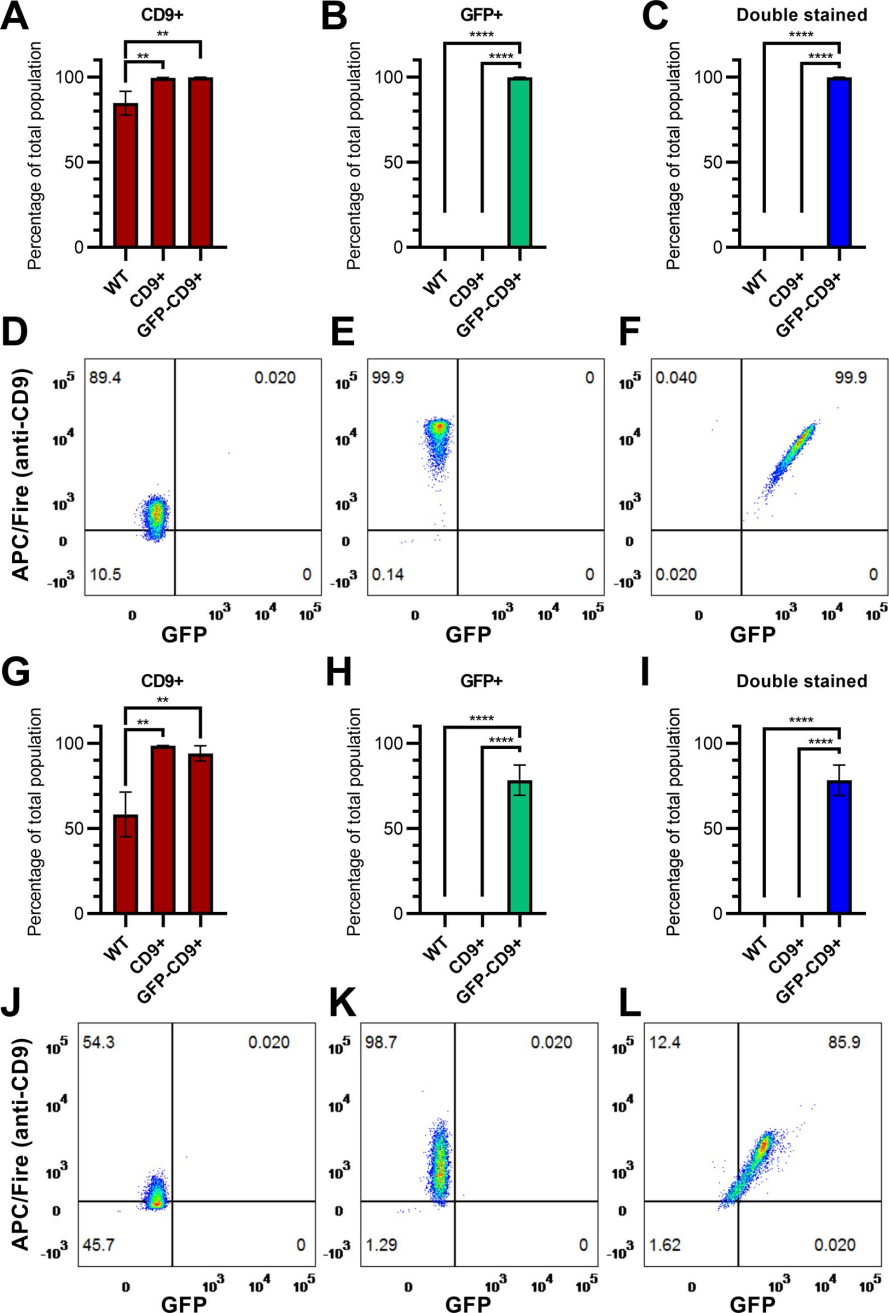
Flow cytometry analysis of EVs. (A) Percentage of CD9+ objects for each cell line after isolation with anti-CD9-beads. (B) Percentage of GFP+ particles after isolation with anti-CD9-beads. (C) Percentage of particles positive for both markers after isolation with anti-CD9-beads. (D) Example of flow cytometry of a EVs sample from WT cells after anti-CD9-beads purification. (E) Example of flow cytometry of a EVs sample from CD9+ cells. (F) Example of flow cytometry of a EVs sample from GFP-CD9+ cells. (G) Percentage of CD9+ particles for each cell line after isolation with anti-CD63-beads. (H) Percentage of GFP+ particles after isolation with anti-CD63-beads. (I) Percentage of particles positive for both markers after isolation with anti-CD63-beads. (J) Example of flow cytometry of a EVs sample from WT cells after anti-CD63-beads purification. (K) Example of flow cytometry of a EVs sample from CD9+ cells. (L) Example of flow cytometry of a EVs sample from GFP-CD9+ cells. Data are represented as mean ± s.d. (n = 3 for each line).

To further confirm increased CD9 expression in our newly generated lines, we selectively isolated CD9+ and CD63+ EVs using commercially available immunofunctionalized beads. Samples were stained with an anti-CD9 antibody and analysed by flow cytometry. After isolation with anti-CD9 beads, there was a significant increase in the percentage of CD9+ particles in samples from CD9+ and GFP-CD9+ cells compared to WT (Fig 2A, WT = 84.75%±7.00 s.d.; CD9+ = 99.61%±0.30 s.d. p = 0.0098; GFP-CD9+ = 99.86%±0.1058 p = 0.0090, Tukey’s multiple comparisons test post ordinary one-way ANOVA). As can be observed in Fig 2D–2F, intensity of CD9-staining was also higher in CD9+ and GFP-CD9+ samples. As expected, no GFP+ particle was detected in samples from WT (0.05%±0.06 s.d.) and CD9+ (0.01%±0.01 s.d.) cells (Fig 2B), while all events recorded in GFP-CD9+ samples were GFP+ (99.81%±0.16 s.d., p<0.0001 compared to the other groups). Similar results were obtained when comparing percentage of double stained particles (Fig 2C, WT = 0.01%±0.012; CD9+ = 0.01%±0.01 s.d.; GFP-CD9+ = 99.80%±0.17 s.d.).

Particles isolated with anti-CD63 beads showed again a significant difference in the percentage of CD9+ events (Fig 2G, WT = 58.27%±13.11 s.d.; CD9+ = 98.54±0.16 s.d.; GFP-CD9+ = 94.10%±4.47 s.d.; WT vs. CD9+ p = 0.0020, WT vs. GFP-CD9+ p = 0.0037). While GFP+ events were detected again only in samples from GFP-CD9+ cells (Fig 2H), their percentage was lower than before (78.38%±8.902 s.d.). The same was observed for double stained particles (Fig 2I, 78.30%±8.97 s.d.). In fact, in Fig 2L a small percentage of events can be seen that were CD9+ but GFP- in EVs from GFP-CD9+ cells.

### Transduction efficiency is not influenced by CD9

**Fig 3:**
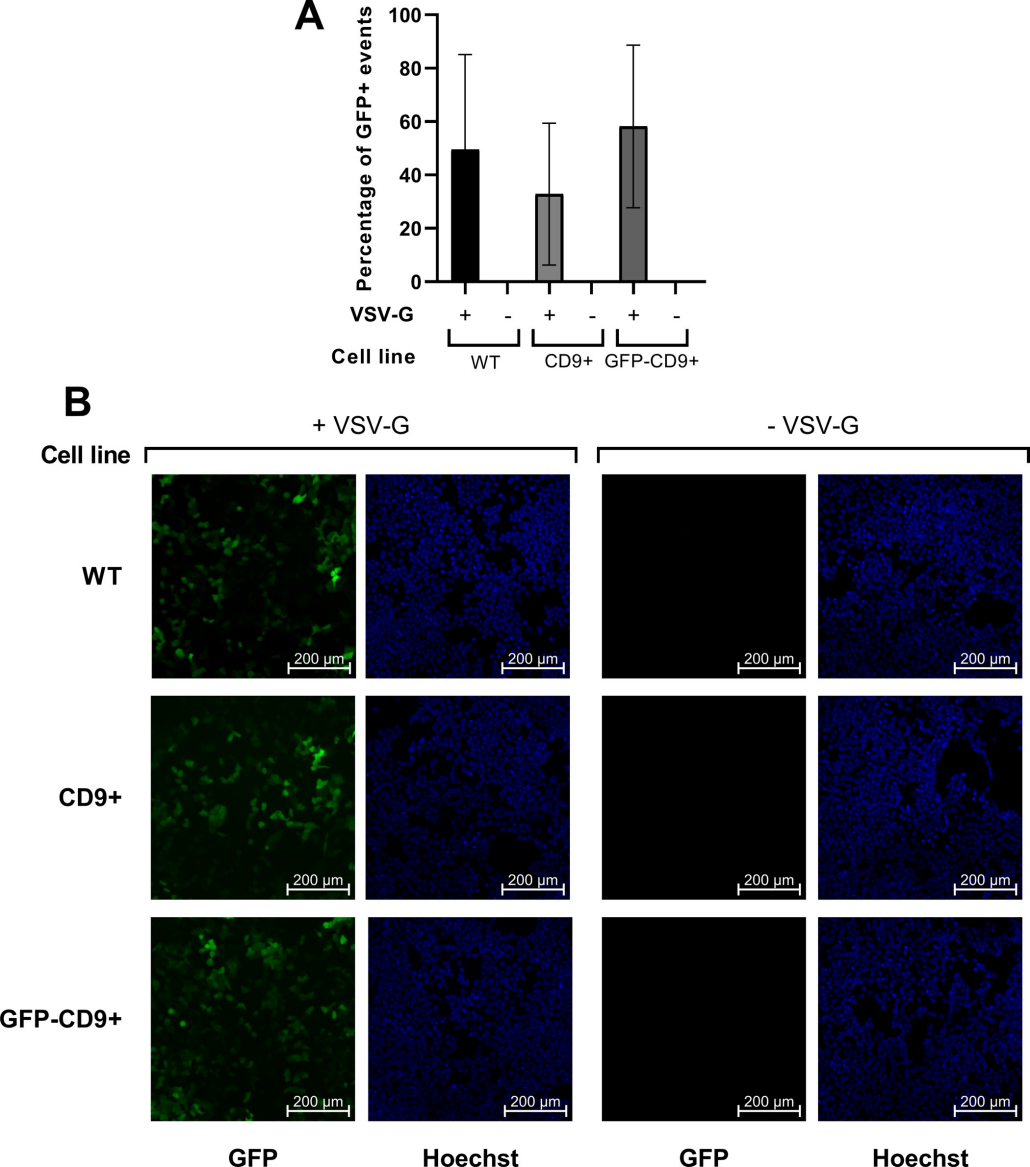
CD9 has no effect on LV transduction. (A) Transduction efficiency of LVs produced in different cell lines ± VSV-G, measured as percentage of GFP+ cells detected by flow cytometry 48h after transduction (n = 5). All data are represented as mean ± s.d. (B) Microscopy images were taken 72h after transduction. GFP signal is shown in green, Hoechst 33342 in blue. No GFP-positive cell was detected in absence of VSV-G (n = 3).

We tested LVs produced in the CD9+ and GFP-CD9+ lines against virus produced in WT cells for differences in transduction efficiency. We found no statistical difference in transduction efficiency assessed by the percentage of GFP positive cells between CD9+ and WT virus samples (Fig 3A; WT 49.51%±35.63 s.d.; CD9+ 32.81%±26.59 s.d.; GFP-CD9+ 58.22±30.46 s.d.; p>0.05 according to Sidak’s multiple comparisons test post Repeated Measures one-way ANOVA). More importantly, we were never able to observe any transduction when VSV-G was not present as a pseudotyping protein on the LVs. In addition, we confirmed our flow cytometry results by fluorescent microscopy (Fig 3B).

### FRα is present on CD9+ and CD63+ EVs

**Fig 4:**
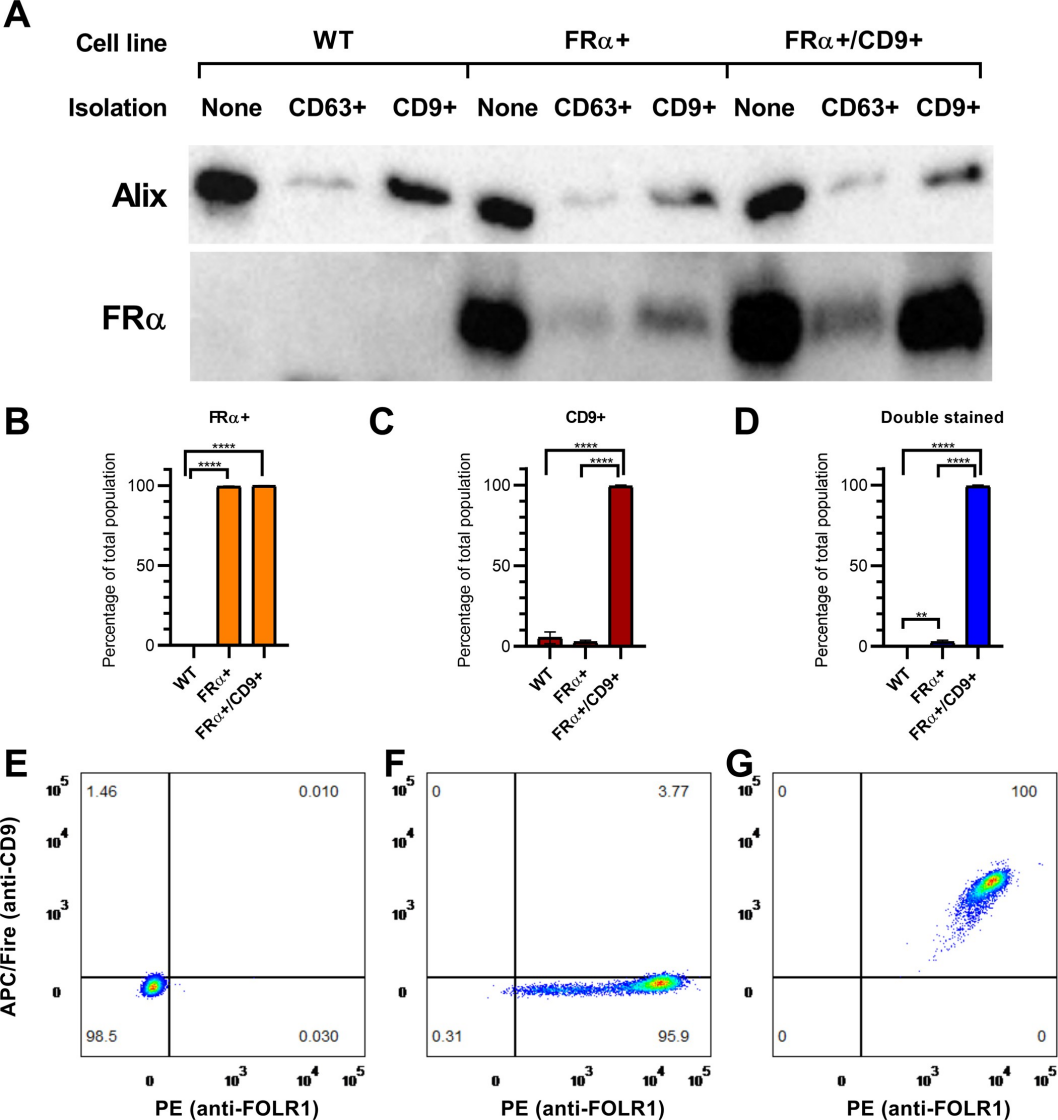
Detection of protein markers in EVs. (A) WB detection of FRα in EVs from WT, FRα+ and FRα+/CD9+ cells after different isolation protocols. Alix was used as a control. For each cell line, 8 μg proteins from EVs before purification with functionalized beads were loaded as reference. (B) Percentage of FRα+ particles for each cell line after anti-CD9-beads purification, measured by flow cytometry. (C) Percentage of CD9+ particles after anti-CD9-beads purification, measured by flow cytometry. (D) Percentage of particles positive for both markers after anti-CD9-beads purification, measured by flow cytometry. (E) Example of flow cytometry of a EVs sample from WT cells after anti-CD9-beads purification. (F) Example of flow cytometry of a EVs sample from FRα+ cells. (G) Example of flow cytometry of a EVs sample from FRα+/CD9+ cells. Data are represented as mean ± s.d. n = 3 for each line.

**Fig 5:**
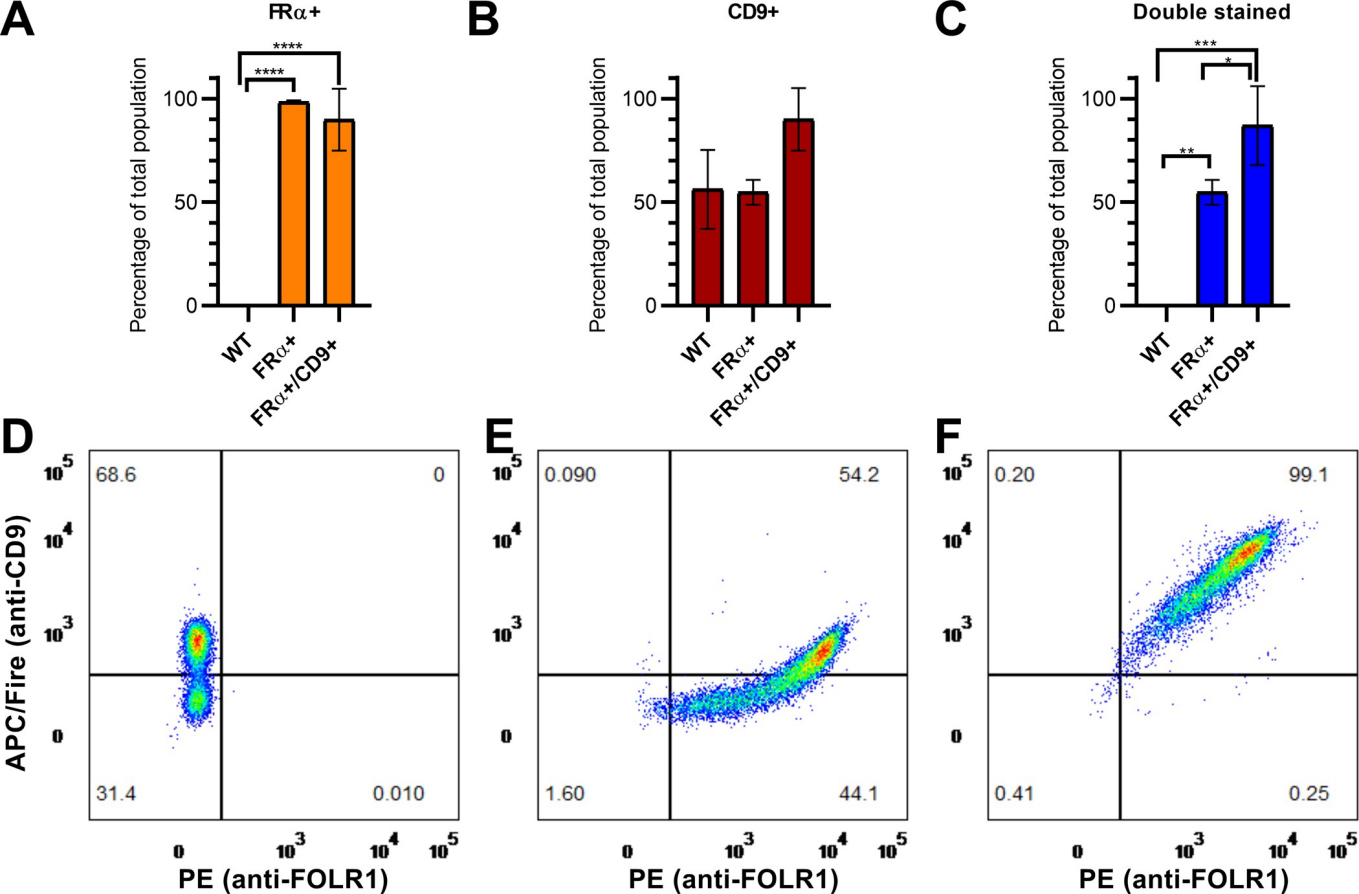
EVs protein markers after isolation with anti-CD63 beads. (A) Percentage of FRα+ particles for each cell line, measured by flow cytometry. (B) Percentage of CD9+ particles, measured by flow cytometry. (C) Percentage of particles positive for both markers, measured by flow cytometry. (D) Example of flow cytometry of a EVs sample from WT cells after anti-CD63-beads purification. (E) Example of flow cytometry of a EVs sample from FRα+ cells. (F) Example of flow cytometry of a EVs sample from FRα+/CD9+ cells. Data are represented as mean ± s.d. (n = 3 for each line). *** = p≤0.001.

At last, we wanted to test if expression of FRα on its own or in combination with CD9 effects LVs transduction efficiency. We first confirmed coexpression of the target proteins on EVs. In fact, small EVs are a heterologous population of cell-derived vesicles: expression of protein markers such as tetraspanins can vary between them [36]. To verify that FRα is not only directed to EVs but is also coexpressed on CD9+ vesicles, we used wild-type HEK 293 (WT) and a previously established cell line overexpressing FRα (FRα+) [35]. Additionally, we generated a cell line overexpressing both FRα and CD9 (FRα+/CD9+ line). Increased expression of the target proteins in the new line was verified by WB (S2 Fig). We then used immunofunctionalized beads to specifically isolate CD9+ or CD63+ EVs from these cells. We tested the obtained EVs populations for the presence of FRα and CD9. FRα was not detectable by WB in EVs obtained from WT cells, but was found in both CD9+ and CD63+ EVs populations from FRα+ and FRα+/CD9+ cells (Fig 4A). Coherently, there was a statistically significant difference between cell lines in staining for FRα when EVs were analysed with flow cytometry. For EVs isolated by using anti-CD9 beads (Fig 4B and 4E–4G), 99.51%±0.26 s.d. of particles from FRα+ cells and 99.96%±0.05 s.d. from FRα+/CD9+ cells were stained for FRα against 0.18%±0.18 s.d. from WT cells (p<0.0001 in both cases, Tukey’s multiple comparisons test post ANOVA). Results were replicated when using anti-CD63 beads for purification (Fig 5A and 5D–5F, p<0.0001 for both WT vs FRα+ and WT vs FRα+/CD9+).

When considering staining for CD9, there was a statistically significant increase (p <0.0001) in EVs from FRα+/CD9+ cells (99.33%±0.70 s.d.) compared to vesicles from the other lines (WT = 5.36%±3.66 s.d.; FRα = 2.78%±1.02 s.d.) after isolation with anti-CD9 beads (Fig 4C). An increase in CD9 staining in EVs from FRα+/CD9+ cells was detected also after isolation using anti-CD63 beads (89.99%±15.07 s.d. vs 56.17%±18.99 s.d. of WT and 54.80%±5.97 s.d. of FRα), albeit it was not statistically significant (Fig 5B).

Finally, looking only at double stained particles, almost all EVs from FRα+/CD9+ cells carried both targets after either isolation protocol (Figs 4D and 5C, anti-CD9 = 99.33%±0.70 s.d., anti-CD63 = 87.00%±19.08 s.d.). This was not observed in the other cell lines (anti-CD9: FRα+/CD9+ vs WT and FRα+/CD9+ vs FRα+ p<0.0001; anti-CD63: FRα+/CD9+ vs WT p = 0.0002, FRα+/CD9+ vs FRα+ p = 0.0327).

### FRα does not influence EVs secretion rate

**Fig 6:**
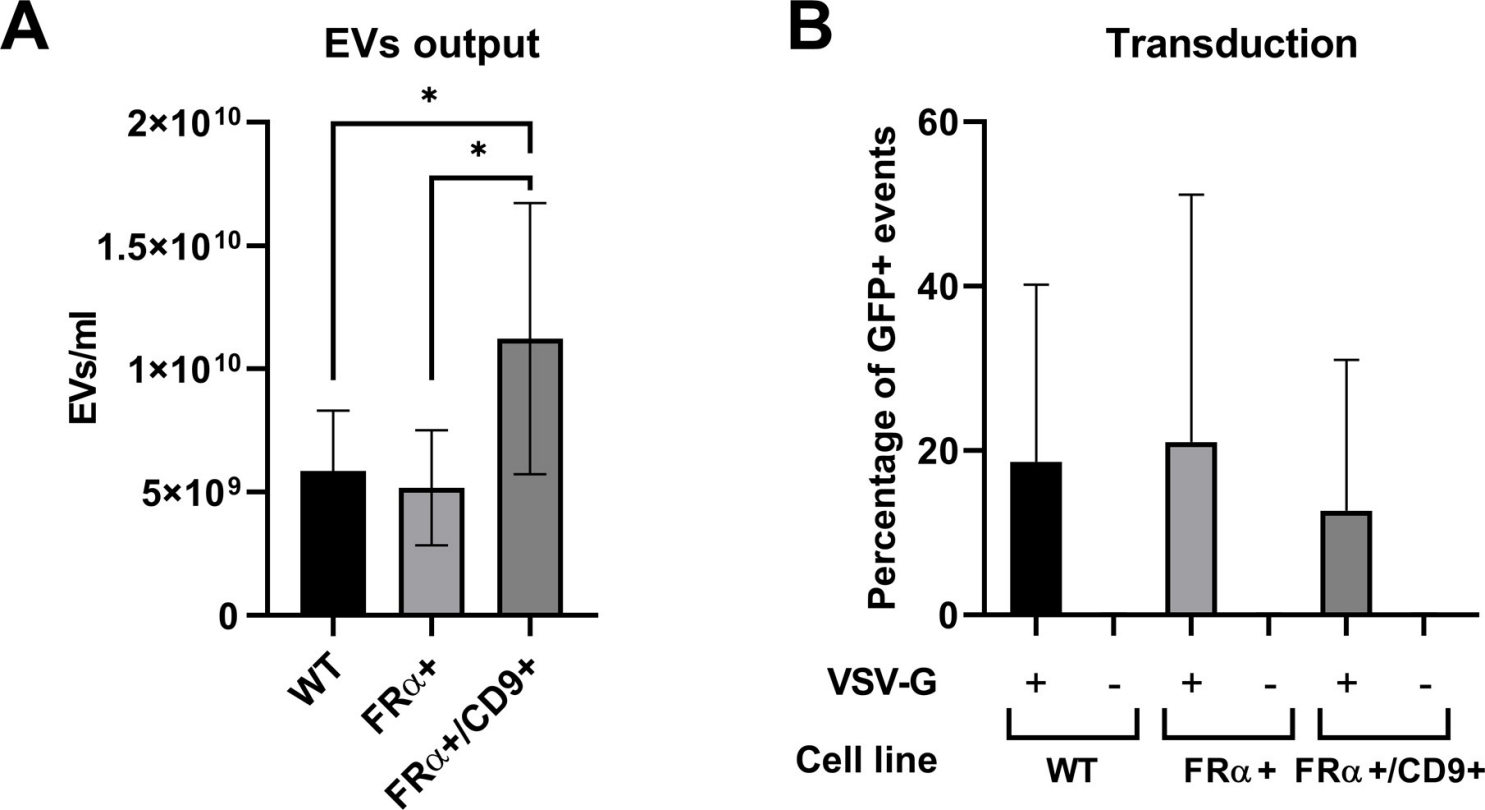
Effect of FRα and CD9 overexpression on EVs production and transduction efficiency. (A) EVs produced by different cell lines over 72h were purified and resuspended in PBS. Concentration was measured by NTA (n = 7). (B) Transduction efficiency of LVs produced in different cell lines, measured as percentage of GFP+ cells detected with flow cytometry 48h after transduction (n = 3). All data are represented as mean ± s.d.

Constitutive expression of CD9 was reported to cause an increase in EVs secretion, while other EVs markers led to a lower EVs output [28]. To check if FRα has an influence on EVs production, we isolated EVs from the WT, FRα+ and FRα+/CD9+ cell lines and measured the sample concentration with Nanoparticle Tracking Analysis (NTA). We found no significant difference in EVs output between WT (5.86*10^9^±2.45*10^9^ s.d. particles/ml) and FRα+ (5.17*10^9^±2.33*10^9^ s.d. particles/ml) samples, indicating that overexpression of FRα per se does not influence EVs secretion (Fig 6A). Coherently with what was previously reported [28], we observed a twofold increase in EVs production in presence of CD9 overexpression (1.12*10^10^ ±5.50*10^9^ s.d. particles/ml, vs WT p = 0.0267, vs FRα+ p = 0.0395, Tukey’s multiple comparisons test post Repeated Measures one-way ANOVA).

### No VSV-G-independent transduction was detectable

To test the effect of FRα and CD9 on lentiviral transduction efficiency, we produced GFP-carrying LVs using either WT, FRα+ or FRα+/CD9+ cells. We assessed the viral transduction by detecting the percentage of fluorescent cells using flow cytometry (Fig 6B). SEW_SFFVU3_GFP was used as a transfer plasmid. We found again no statistically significant difference in transduction efficiency between viruses produced in either cell line (Sidak’s multiple comparisons test post Repeated Measures one-way ANOVA, WT 18.62%±21.54 s.d., FRα+ 21.04%±30.06 s.d., FRα+/CD9+ 12.70%±18.32 s.d.). Moreover, when VSV-G was not present as envelope protein, no transduction at all was observed, independently of the expression levels of CD9 (see also S3 Fig).

## Discussion

Our study aimed to identify mammalian proteins that are able to mediate transduction of lentiviral particles when expressed in the viral envelope. Two possible candidate proteins, CD9 and FRα, were investigated. At first we wanted to replicate results previously reported by Boker and colleagues concerning CD9-mediated VSV-G-independent viral transduction. We established two HEK 293T CD9 overexpressing cell lines following closely their protocol. Flow cytometry characterization of EVs isolated from these lines confirmed the increase of CD9 expression in both CD9+ and CD63+ EVs populations. Surprisingly, when anti-CD9 beads were used for the isolation, a small percentage of particles in the WT samples were not stained for CD9. Since CD9 was used to isolate these samples, all particles should be positive for this marker. A possible explanation is that antibodies attached to the beads may compete with fluorescently labelled anti-CD9 antibodies for CD9 binding sites. This would suggest the presence of a gradient of CD9 expression in WT EVs. When anti-CD63 beads were used, around 58% of the WT particles were stained CD9+, indicating the presence of CD9- EVs populations. In addition, analysing GFP-CD9+ samples we observed a small CD9+/GFP- population. This can be explained by discrepancies in sorting of native CD9 and GFP-CD9 fusion protein in CD63+ EVs populations, but it could also be caused by a difference in the strength of the antibody signal compared to the fluorescent protein.

We used our newly established lines to produce CD9-enriched LVs. Unexpectedly, our experiments showed that in absence of VSV-G, there was no detectable transduction. In addition, we failed to record an increase in transduction efficiency when VSV-G was present, even if our protocols followed closely the ones previously reported [28].

Further, we investigated if FRα was able to affect the transduction efficiency, on its own or in combination with CD9. Therefore, we established a FRα/CD9 overexpressing line starting from HEK 293.

In our previous study, we have demonstrated that FRα-expressing EVs can be selectively isolated from the cell culture supernatant [35]. However, it was possible that FRα was only expressed by a restricted subpopulation of EVs, thus rendering it less efficient for EVs isolation then other more general markers. In this study, we demonstrated that, when constitutively expressed, FRα is carried by CD63+ and CD9+ EVs, which are traditionally considered EVs markers [29, 37]. The fact that samples from the FRα+ and FRα+/CD9+ cell lines were positively stained for FRα independently from the type of functionalized beads that were used, confirms that FRα is coexpressed with established EVs markers.

Concerning the flow cytometry results, it is interesting to note that there is a major difference between the number of CD9+ events in WT EVs when the sample was isolated using either anti-CD63 or anti-CD9 beads. When anti-CD63 beads were used, around 56% of the WT particles were stained CD9+, coherently with what we observed in the WT HEK 293T.In contrast, when anti-CD9 beads were used, only a minimal percentage of WT EVs were positively stained for CD9, independent of the expression of FRα. This could suggest that the majority of EVs produced by WT HEK 293 express lower levels of CD9 than EVs derived from WT HEK 293T. In contrast, when CD9 was overexpressed in our FRα+/CD9+ line, almost all detected particles were stained for it independently of the type of beads that were used to purify the samples. This indicates that overexpression of CD9 results in the presence of CD9 in the membrane of EVs.

FRα also failed to affect the LVs transduction efficiency, even in combination with high levels of CD9 expression.

Our results indicate that overexpression of CD9 on its own is not sufficient to increase transduction efficiency, unlike the results obtained previously [28]. In their paper, Boker and colleagues argued that the enhanced number of EVs secreted by CD9-enriched cells was not the key factor in increasing the LVs transduction efficiency, since adding external exosomes to the LVs had a negative effect on transduction. Our results confirmed the CD9-dependent rise in EVs production but failed to demonstrate any increase in LVs transduction efficiency. The discrepancies in transduction competence could be explained by several factors. Although we tried to strictly reproduce their protocols, some experimental factors still differed. First of all, we used a different packaging plasmid for LVs production: psPAX2 and pCMV-dR8.91 are both second generation plasmids and share similar architecture and gene compositions [38], but there could be differences in efficiency of virus production. For example, a recent study showed that pCMV-dR8.2 dvpr was able to stimulate a lentivirus production at least 7 times stronger than psPAX2 [39].However, since we always used the same titre of virus for transduction, this should not affect transduction efficiency per se. Further, the cell line used to produce the LVs might determine several characteristics of the EVs. The protein composition of EVs depends on the secreting cell type [40] and one cell type may produce different subpopulations of EVs [41]. EVs composition also changes in pathological conditions and thus can be exploited as potential diagnostic biomarkers, mostly in cancer [42] but also in other pathological conditions [43]. While we overexpressed CD9 and GFP-CD9 in HEK 293T cells, Boker and colleagues used the HEK 293FT line. For the establishment of FRα expressing cells, we used HEK 293 cells. The major difference between HEK 293T and HEK 293FT is the faster metabolism and therefore enhanced growing rate of the latter one. Both lines express the SV40 large T-Antigen that is not present in the original HEK 293 line. The faster metabolism of HEK 293FT might lead to a higher LV release rate. However, since we used the same virus titre for transduction this should not matter. Possibly, some particular characteristic of the EVs secreted by HEK 293FT could be present in combination with CD9 overexpression to mediate transduction. Our flow cytometry results suggest a difference in CD9 expression on EVs derived either from WT HEK 293 and or from HEK 293T cells, as discussed above. Consequently, we assume that even cell lines that are as close as HEK 293, 293T and 293FT can show differences in their EVs composition.

Another difference between our experiments and the ones of the reference study could be found in the levels of CD9 expression that were obtained in the stable cell lines. When looking at qPCR results, we obtained a much bigger increase in CD9 expression than the previous study using the pLenti6.3-CD9_GFP_ plasmid (576-fold increase versus 22-fold). On the other hand, we verified by WB that the CD9 expression did only slightly increase. This discrepancy between mRNA and proteins levels has recently been substantiated by omics studies indicating that protein abundance only partly correlates with mRNA increase [44]. The amount of CD9 that is actually translated could be limited by the cells on a post-transcriptional level. For example, it is known that miR-518f-5p decreases CD9 levels in some types of cancer cells [45, 46]. Moreover, the fact that similar increases in mRNA synthesis between the CD9+ and GFP-CD9+ lines were associated with significant differences in CD9 protein expression suggests that the fusion protein is subjected to distinct post-transcriptional regulation and metabolic degradation in comparison to WT CD9. It might therefore be misleading to make comparisons between cell lines only based on qPCR data. It is theoretically possible that different cell lines tolerate different levels of CD9 to be translated and that VSV-G-independent transduction can happen only when a certain level of CD9 is reached and/or coincides with the expression of other proteins.

Finally, it is possible that Boker and colleagues did not observe proper transduction events. If we consider that their GFP-CD9+ line had a higher EVs secretion rate, but the obtained lentivirus titre was the same as the one from WT cells, we can hypothesize that these cells produced an excess of non-lentiviral EVs. It is also possible that a higher EVs secretion rate could increase the likelihood of cytosolic proteins like GFP and RFP to be passively loaded into EVs. Those vesicles would have been collected and concentrated together with the virus. Since a constant virus titre was used in transduction experiments, only cells transduced with the GFP-CD9+ viruses would have received a higher number of EVs. Consequently, it’s possible that CD9, increasing EVs production, had the effect of increasing the probability of EV-mediated delivery of the fluorescent protein without any direct effect on transduction efficiency. Boker and colleagues did not report the exact percentage of transduced cells they observed when VSV-G was omitted, but they stated that it was “a minor proportion”. That would be in line with recent reports demonstrating that EV-cargo-delivery is very inefficient [47]. Since we always obtained very high virus titres we directly used the cell supernatant for transduction experiments. It is possible that we did not apply enough EVs carrying fluorescent proteins to observe fluorescent cells. Another possibility is that some vesicle degradation occurred during the virus concentration step, releasing the fluorescent proteins contained in EVs. Once again, since virus samples from the GFP-CD9+ cell line would contain more EVs, they would potentially carry more contaminants. Cases of pseudotransduction caused by co-purified protein during the concentration step were observed in the past [48]. Since we did not perform a concentration step, we have avoided this kind of pseudotransduction.

In conclusion, we confirmed that the mechanism of increase in EVs production depends on CD9 expression. On the other hand, we could neither confirm a function of CD9 as viral-independent transduction mediator nor demonstrate a positive effect of FRα on the transduction efficiency. Our results confirm recent studies reporting that only EVs modified with a fusogenic protein such as VSV-G could deliver their cargo to cells at detectable levels [49, 50]. Moreover, even when rare cargo-delivery events could be detected with a CRISPR/Cas9 reporter system, EV-mediated RNA transfer was not observed when HEK 293T cells were used as EVs donor [47].

Although we did not achieve the hoped results in this study, we remain convinced of the potential held by the expression of EVs markers on LVs surface for transduction. Until now, strategies for LVs pseudotyping only focused on viral glycoproteins from different viruses [51]. The same proteins (or portions of them) have also been expressed on the surface of EVs in the attempt to increase specific targeting, for example to the brain [52]. On the other hand, EVs are meant to be taken up by cells and some of them show preferred targeting to certain cell types or tissues. As we discover more and more about the mechanisms that endogenously regulate EVs uptake and targeting [53], new candidate proteins may emerge. Expression of endogenous markers instead of immunogenic viral proteins on LVs surface has the potential of opening the way to a new class of gene therapy vectors with increased safety and efficacy.

## Supporting information

S1 Table List of primers. (PDF)

S1 Fig GFP-CD9 expression in stable cell line. Cells from the GFP-CD9+ cell line were stained with Hoechst 33342 1:2,000 (in blue). Images were taken with a 40X objective. Expression of the fusion protein can be seen located to the cell membrane.
(TIF)

S2 Fig Expression of FRα and CD9 in FRα+/CD9+ line compared to WT. HSP90 was used as loading control.
(TIF)

S3 Fig Example of transduction with LVs produced in WT, FRα+ or FRα+/CD9+ cells. Images were taken 72h after transduction. GFP signal is shown in green, Hoechst 33342 in blue. No GFP-positive cell was detected in absence of VSV-G.
(TIF)

S1 File Original blot images. (PDF)
