# Supplementary material for: CD9 and folate receptor overexpression are not sufficient for VSV-G-independent lentiviral transduction
Source: PLoS One. 2022 Mar 10;17(3):e0264642. doi: 10.1371/journal.pone.0264642 (PMC8912258; doi:10.1371/journal.pone.0264642)
Supplement: S1 Table — (PDF) [file pone.0264642.s001.pdf]

Primer name	Function	Sequence
CD9_mRNA_for	Primer to amplify CD9 mRNAS from cDNA	ATC TGT ATC CAG CGC CAG GT
CD9_mRNA_rev	Primer to amplify CD9 mRNA from cDNA	TCC TGC TCA GGG ATG TAA GC
NheI-CD9_for	Primer to attach restriction site to CD9	TAA GCT AGC CCT CAC CAT GCC GG
XhoI-CD9-rev	Primer to attach restriction site to CD9	ATG ACT CGA GAC TCT AGA CCA TCT CGC GG
CD9_seq_for	Sequencing primer internal to CD9	ATT CCA GCT TCT ACA CAG GAG
CD9_seq_rev	Sequencing primer internal to CD9	GAC TCT AGA CCA TCT CGC GG
FR1_seq_for	Sequencing primer internal to FRα	GAC ACC TGC CTC TAC GAG TG
B2M_for	qPCR primer	TGT GCT CGC GCT ACT CTC TCT
B2M_rev	qPCR primer	CGG ATG GAT GAA ACC CAG ACA
hCD9_for	qPCR primer	ATG ATG CTG GTG GGC TTC
hCD9_rev	qPCR primer	GCT CAT CCT TGG TTT TCA GC
