# Supplementary figures and images for: CD9 and folate receptor overexpression are not sufficient for VSV-G-independent lentiviral transduction
Source: PLoS One. 2022 Mar 10;17(3):e0264642. doi: 10.1371/journal.pone.0264642 (PMC8912258; doi:10.1371/journal.pone.0264642)

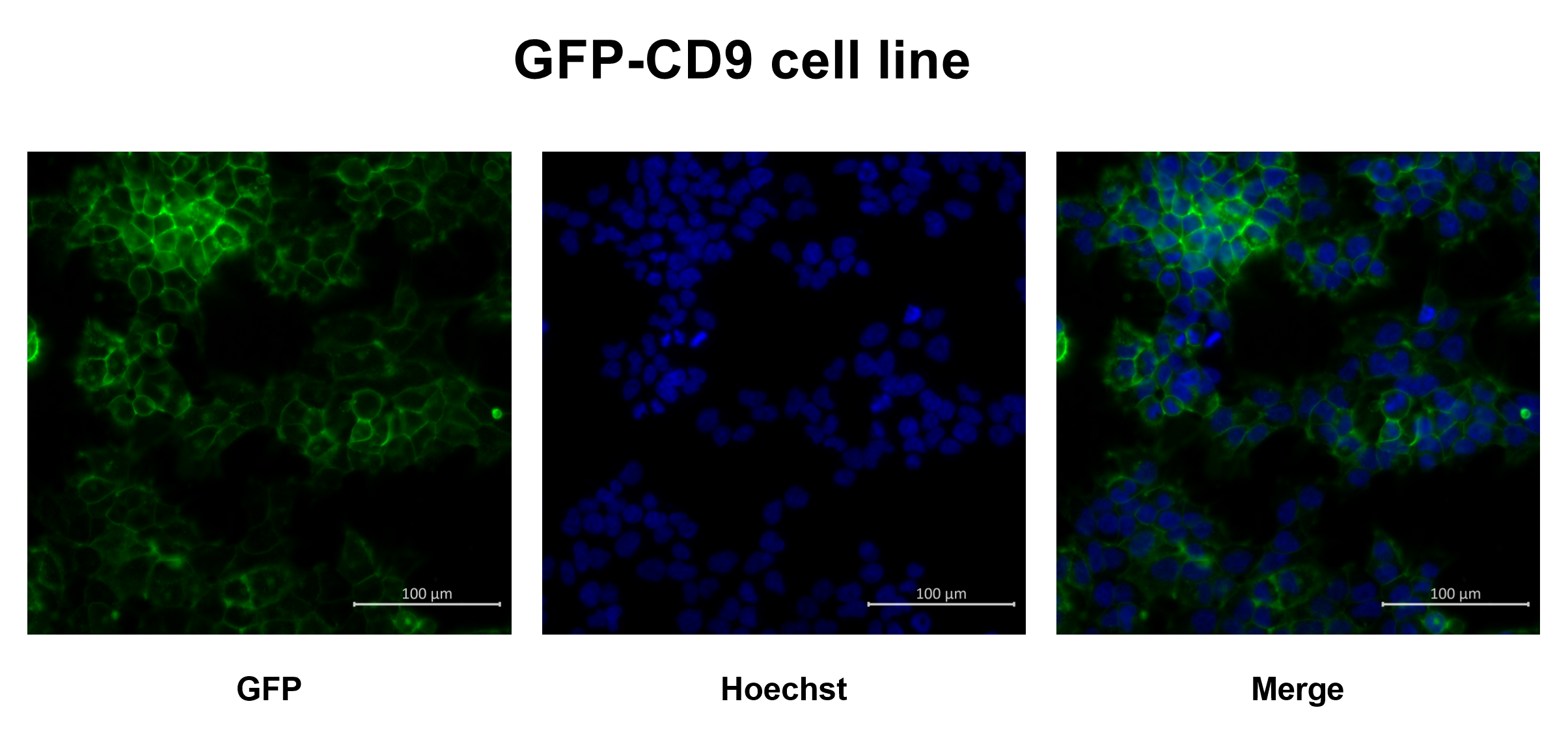

Supplement: S1 Fig — Cells from the GFP-CD9+ cell line were stained with Hoechst 33342 1:2,000 (in blue). Images were taken with a 40X objective. Expression of the fusion protein can be seen located to the cell membrane. (TIF) [file pone.0264642.s002.tif]

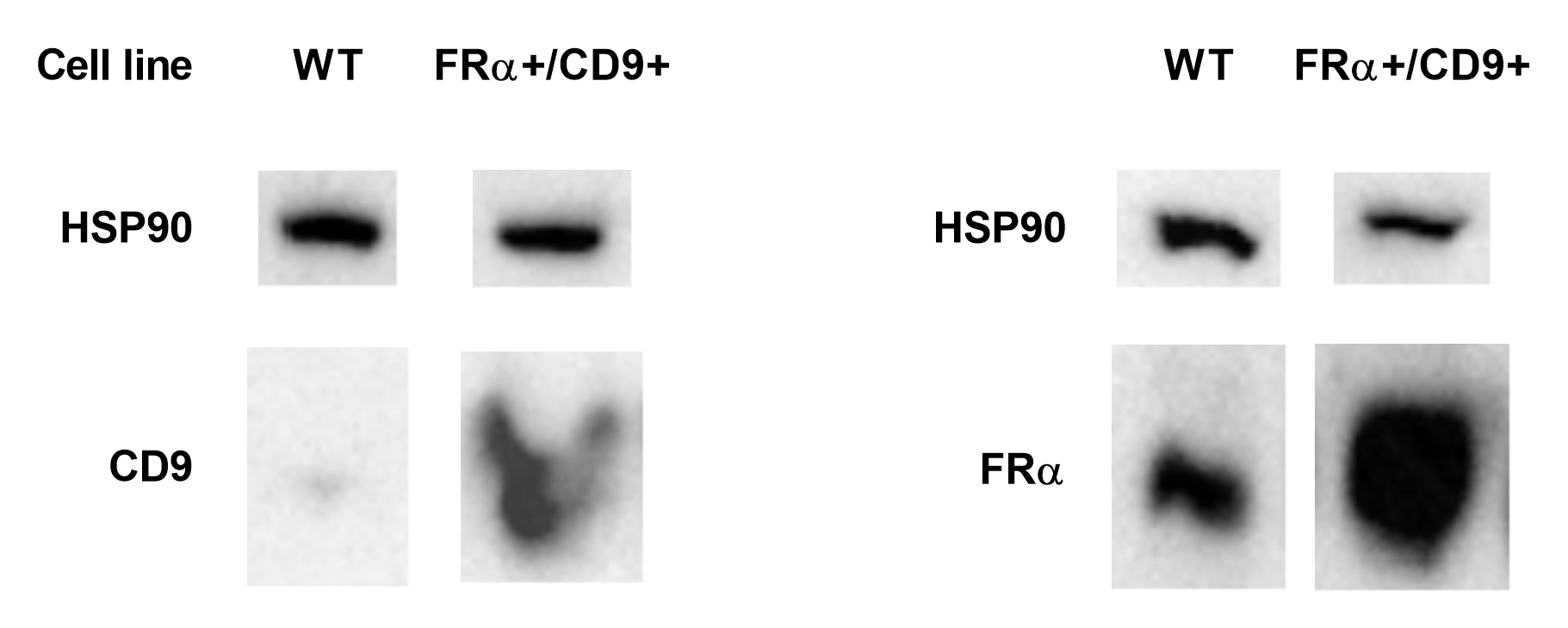

Supplement: S2 Fig — HSP90 was used as loading control. (TIF) [file pone.0264642.s003.tif]

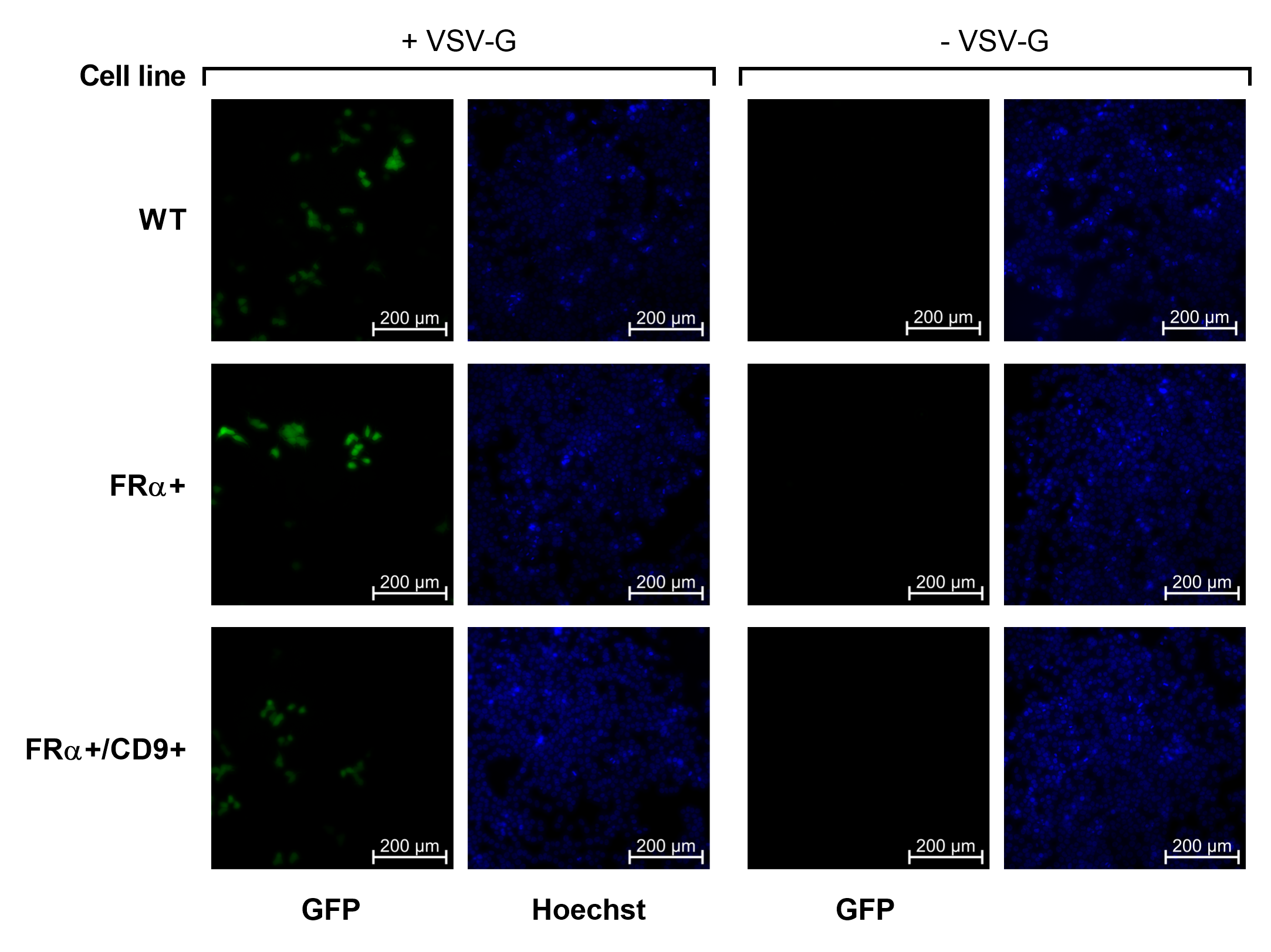

Supplement: S3 Fig — Images were taken 72h after transduction. GFP signal is shown in green, Hoechst 33342 in blue. No GFP-positive cell was detected in absence of VSV-G. (TIF) [file pone.0264642.s004.tif]
