# Supplementary material for: CD9 and folate receptor overexpression are not sufficient for VSV-G-independent lentiviral transduction
Source: PLoS One. 2022 Mar 10;17(3):e0264642. doi: 10.1371/journal.pone.0264642 (PMC8912258; doi:10.1371/journal.pone.0264642)

Blot presented in Fig 1.
Staining: anti-HSP 90α/β + Goat Anti-Mouse IgG H&L (HRP-conjugated).
Exposure: 3.3 sec

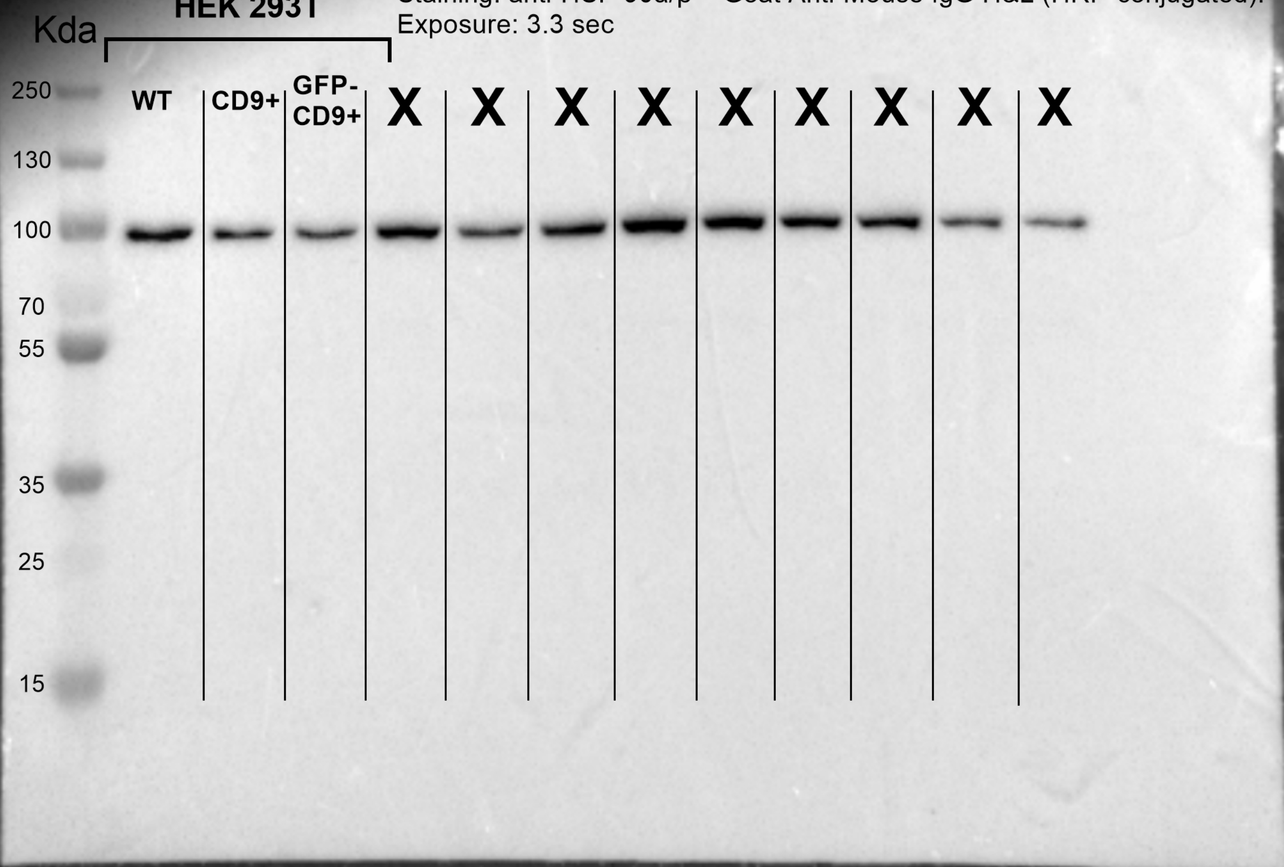

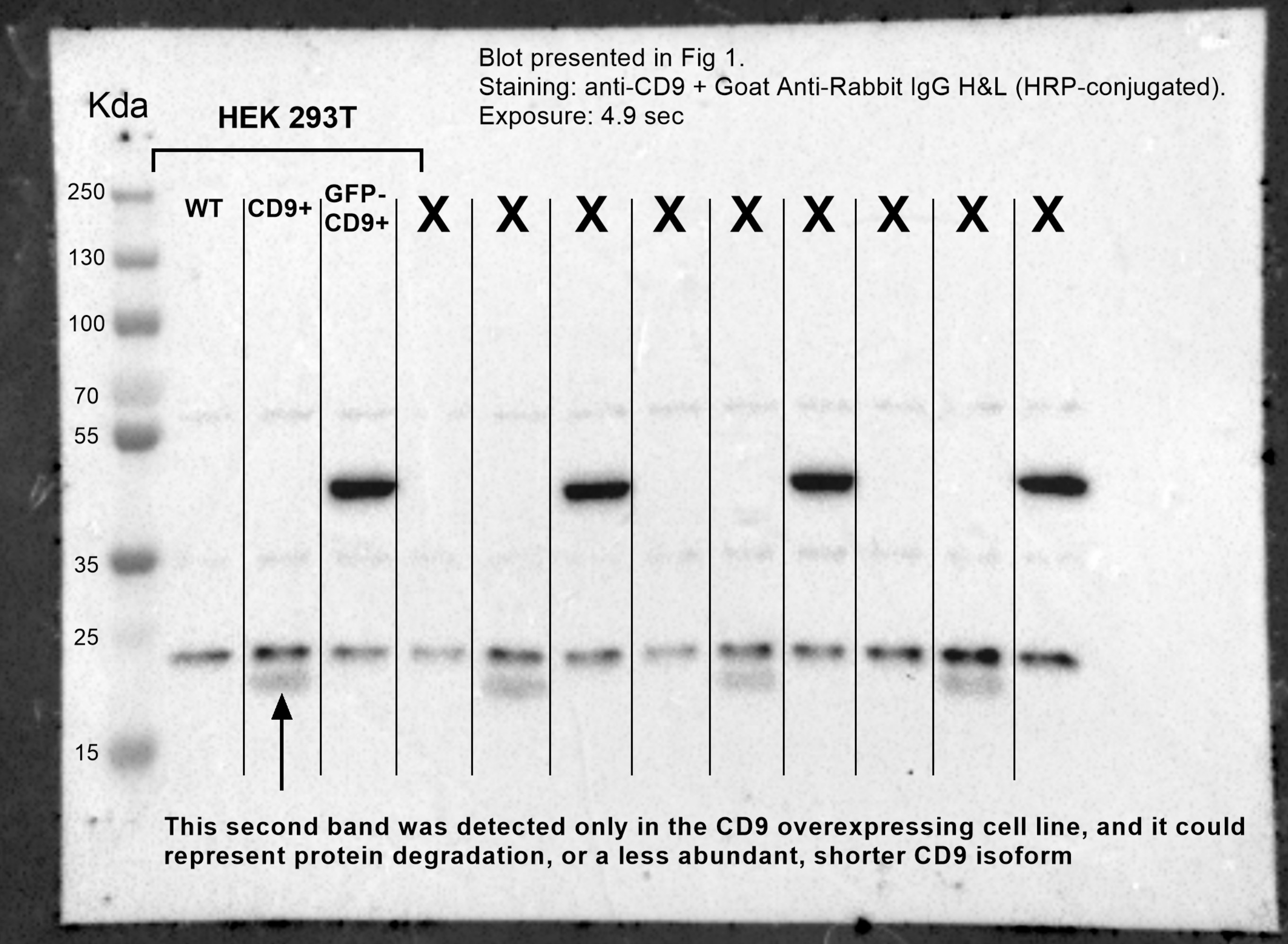
Blot presented in Fig 1.
Staining: anti-CD9 + Goat Anti-Rabbit IgG H&L (HRP-conjugated).
Exposure: 4.9 sec
Kda
HEK 293T
250
130
100
70
55
35
25
15
WT
CD9+
GFP-CD9+
X
X
X
X
X
X
X
X
X
This second band was detected only in the CD9 overexpressing cell line, and it could represent protein degradation, or a less abundant, shorter CD9 isoform

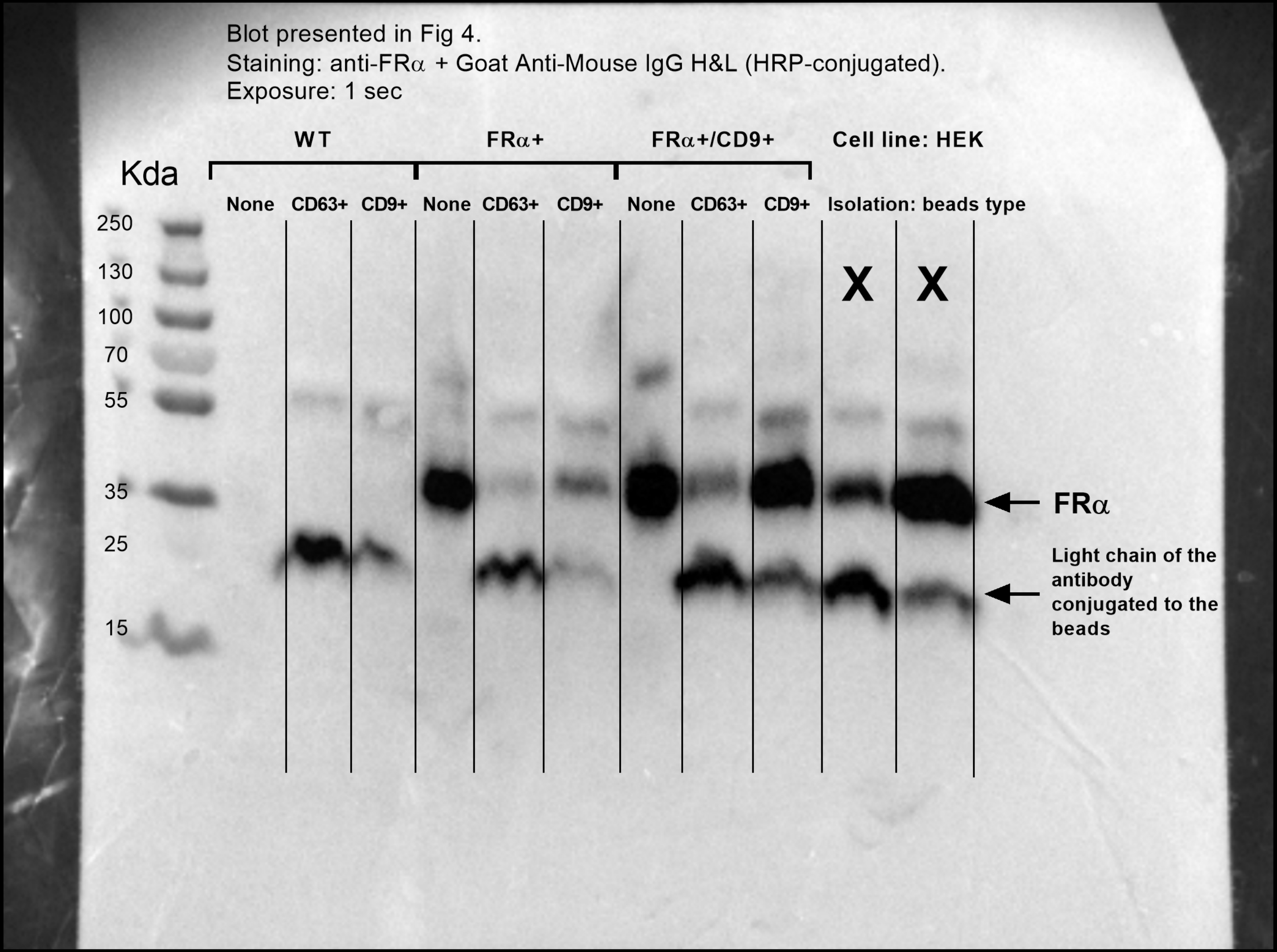
Blot presented in Fig 4.
Staining: anti-FRα + Goat Anti-Mouse IgG H&L (HRP-conjugated).
Exposure: 1 sec
WT
FRα+
FRα+/CD9+
Cell line: HEK
Kda
None
CD63+
CD9+
None
CD63+
CD9+
None
CD63+
CD9+
Isolation: beads type
250
130
100
70
55
35
25
15
X
X
FRα
Light chain of the antibody conjugated to the beads

Blot presented in Fig 4.
Staining: anti-Alix + Goat Anti-Rabbit IgG H&L (HRP-conjugated).
Exposure: 10 sec

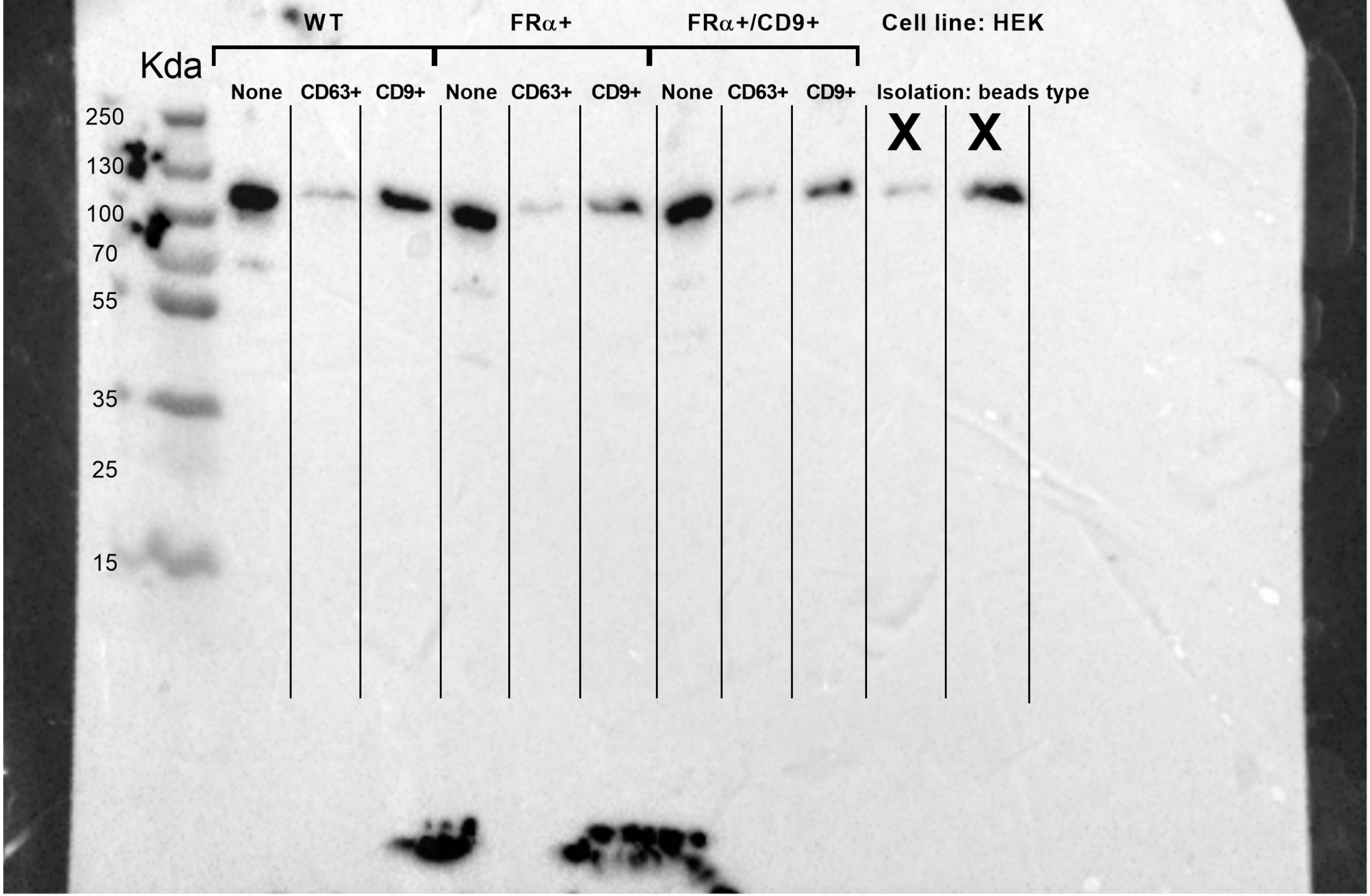

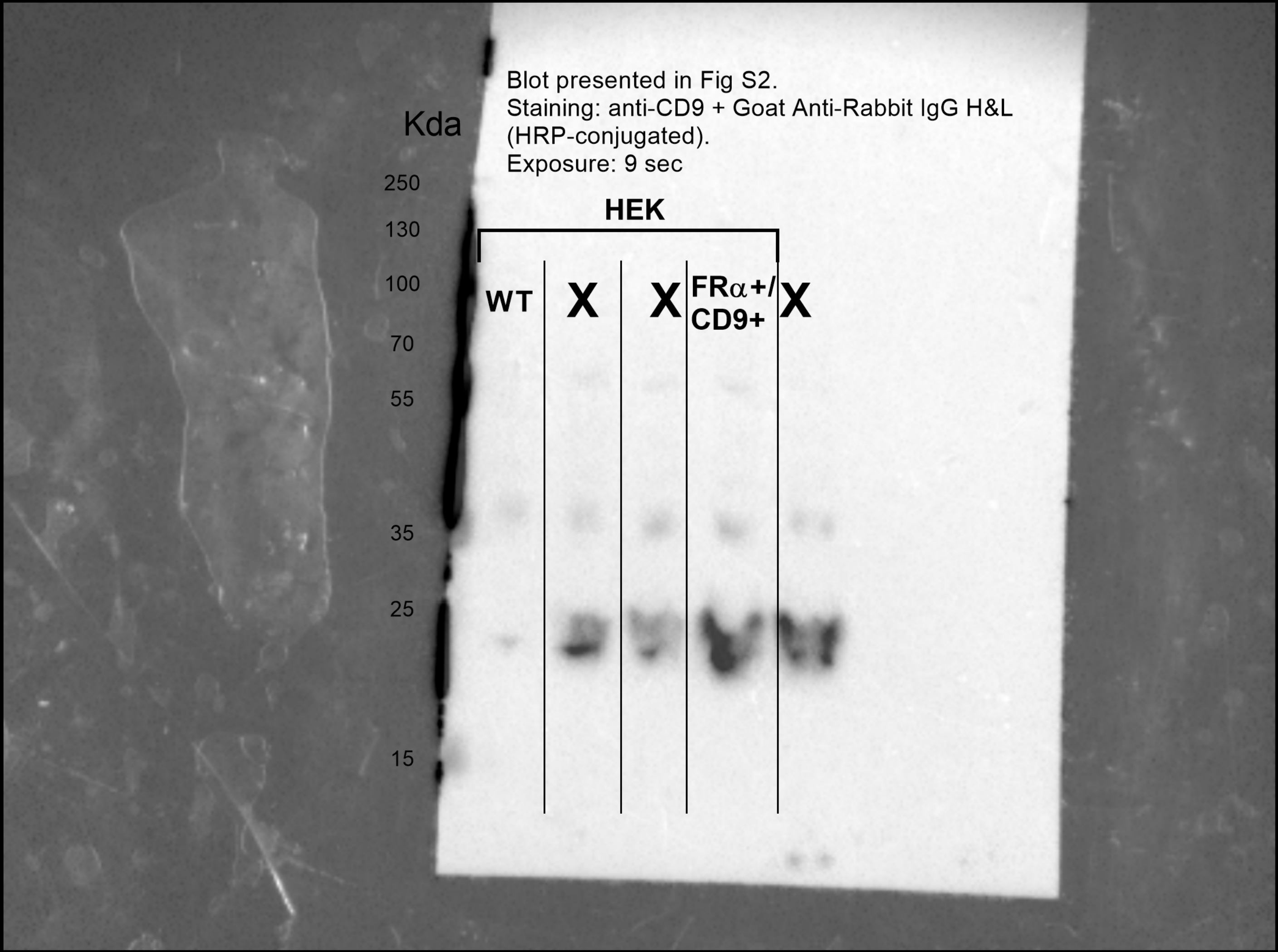
Blot presented in Fig S2.
Staining: anti-CD9 + Goat Anti-Rabbit IgG H&L (HRP-conjugated).
Exposure: 9 sec
Kda
250
130
100
70
55
35
25
15
HEK
WT
X
X
FRα+/CD9+
X

Blot presented in Fig S2.
Staining: anti-FRα + Goat Anti-Mouse IgG H&L (HRP-conjugated).
Exposure: 1.9 sec

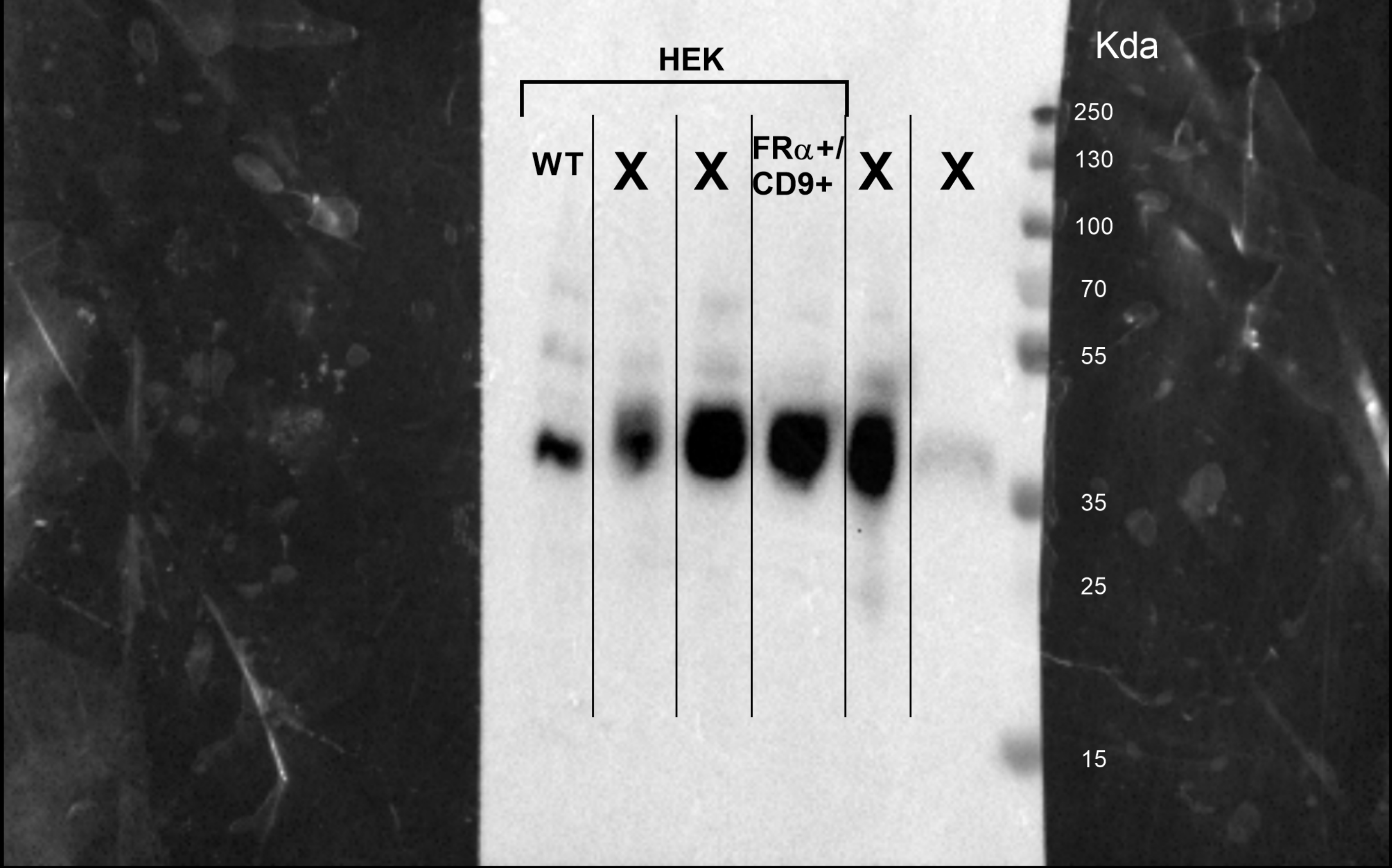

Blot presented in Fig S2.
Staining: anti-HSP 90α/β + Goat Anti-Mouse IgG H&L (HRP-conjugated).
Exposure: 1.4 sec

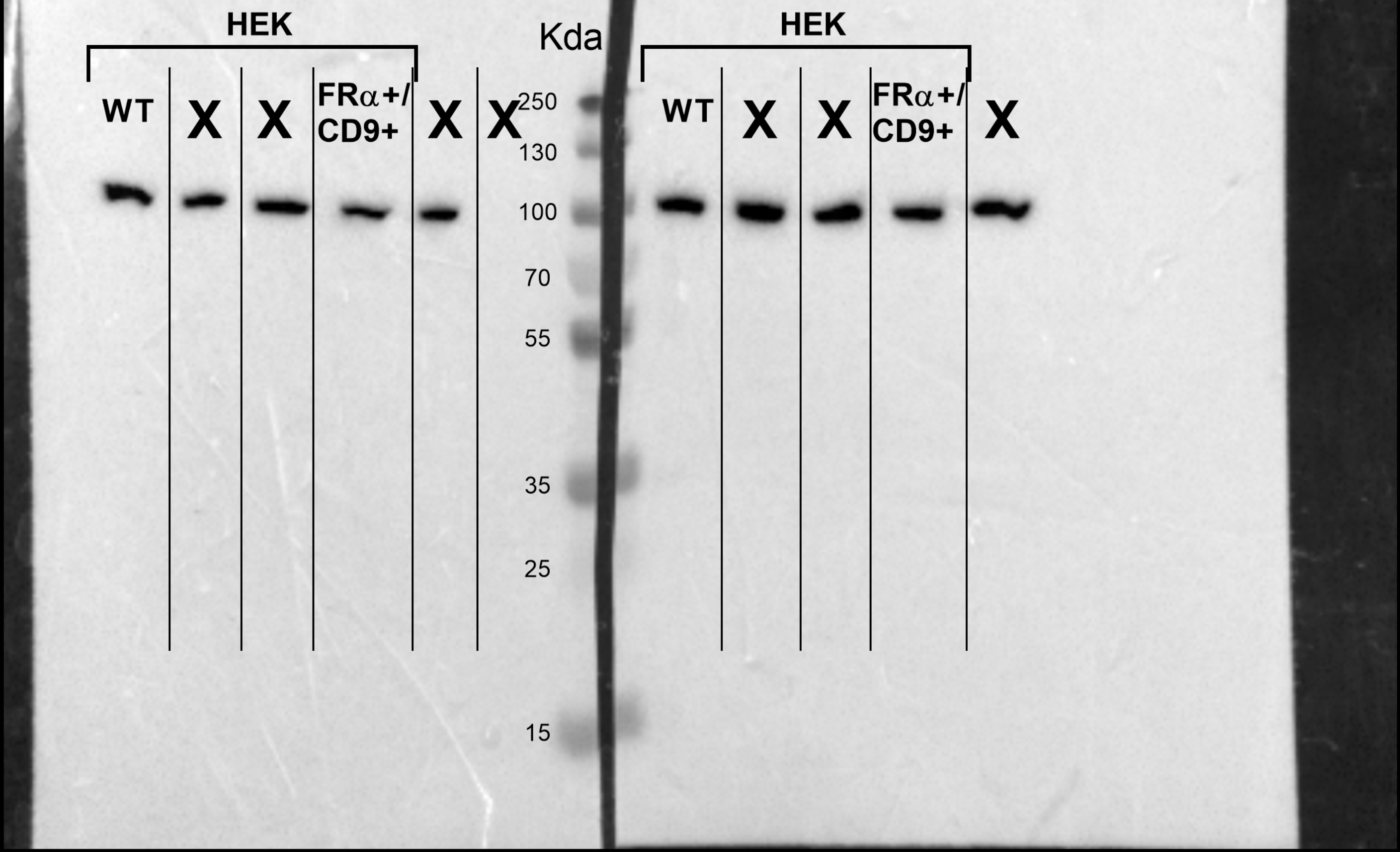

Supplement: S1 File — (PDF) [file pone.0264642.s005.PDF]
